# BDNF-driven synaptic plasticity requires autocrine matrix metalloproteinase–9 activity

**DOI:** 10.1126/sciadv.adx2369

**Published:** 2025-09-24

**Authors:** Diana Legutko, Lukasz Bijoch, Grzegorz Olszak, Bożena Kuźniewska, Katarzyna Kalita, Ryohei Yasuda, Leszek Kaczmarek, Piotr Michaluk

**Affiliations:** ^1^ BRAINCITY, Laboratory of Neurobiology, The Nencki Institute, Pasteura 3, 02-093 Warsaw, Poland.; ^2^ Max Planck Florida Institute for Neuroscience, 1 Max Planck Way, Jupiter, FL 33458, USA.

## Abstract

Structural plasticity of dendritic spines is a fundamental mechanism of learning and memory. It depends on the release of brain-derived neurotrophic factor (BDNF) and activation of its receptor, tropomyosin receptor kinase B (TrkB). However, to bind TrkB, BDNF requires proteolytic cleavage to its mature form. Here, we demonstrate that MMP-9, an extracellular protease essential for neuronal function, plays a key role in this process. We show that, like BDNF, MMP-9 is rapidly released in response to synaptic stimulation, and its proteolytic activity, restricted to the activated spine, can be detected as early as 2 min after stimulation. Using two-photon microscopy and single-spine stimulation by glutamate uncaging, we demonstrate that MMP-9 action is important for TrkB activation and is required for structural plasticity. Furthermore, we provide evidence for a direct cleavage of proBDNF into mature BDNF by MMP-9. Our findings reveal a critical interaction between MMP-9 and BDNF through their autocrine regulation of TrkB activation and dendritic spine structural changes.

## Teaser

Extracellular protease, MMP-9, controls structural synaptic plasticity by regulating TrkB activation at a single synapse.

## INTRODUCTION

Synaptic plasticity is the ability of neuronal connections to either strengthen or weaken over time. It is a fundamental process underlying many brain functions, including learning and memory. Aberrant synaptic plasticity has also been implicated in several neurological and psychiatric disorders, such as schizophrenia, autism spectrum disorders, addiction, depression, and epilepsy. Synaptic plasticity has been extensively studied, mainly on excitatory synapses located on small neuronal protrusions called dendritic spines. Dendritic spine size corresponds to synaptic strength, which reflects the amplitude of excitatory postsynaptic current (*1*, *2*). One of the manifestations of synaptic plasticity is a structural change in dendritic spine volume, which markedly increases upon repetitive glutamate stimulation (*3*). Multiple molecular pathways regulating synaptic plasticity have been identified (*4*). Many extracellularly operating and transmembrane elements of these pathways include such proteins as cell adhesion molecules, components of extracellular matrix (ECM), neurotrophic factors [with brain-derived neurotrophic factor (BDNF) being the most prominent], and proteolytic enzymes [including matrix metalloproteinases (MMPs)] (*4*, *5*).

Since its discovery, the role of BDNF in the synaptic regulation of brain physiology and pathology has attracted a lot of attention (*6*). However, due to inconsistent findings and complex underlying mechanisms of its action, studies have tempered optimism regarding BDNF’s role as a straightforward therapeutic target (*6*). BDNF is synthesized in a precursor form (proBDNF) and is proteolytically processed to its mature form (mBDNF; C-terminal) and a propeptide (N-terminal). Most proBDNF is cleaved intracellularly either by furin in the trans-Golgi network or by proprotein convertases 1/3/7 in secretory vesicles, leading to the release of mBDNF together with the propeptide (*7*–*11*). mBDNF can be released either pre- or postsynaptically. Its receptor, tropomyosin receptor kinase B (TrkB), is also found on both sides of the synapse. Postsynaptic TrkB receptors activated by mBDNF have been implicated in the induction of long-term potentiation (LTP), a phenomenon widely regarded as a model of synaptic plasticity (*12*). Furthermore, both pre- and postsynaptic TrkB receptors are believed to be mainly involved in LTP maintenance (*13*). A smaller fraction of proBDNF can also be released directly to the extracellular space (*8*–*11*, *14*). Once released, proBDNF binds to p75^NTR^ receptor, facilitating long-term depression (LTD) (*15*), or leads to apoptosis by forming a complex with p75^NTR^ and sortilin (*16*). The cleaved-off BDNF propeptide can also activate the p75^NTR^ receptor and facilitate LTD (*17*), and lead to a reduction of dendritic spine density (*18*). Finally, released proBDNF can also be processed to mBDNF extracellularly by proteases through a complicated cascade of enzymes and their inhibitors (*19*–*23*), including plasminogen, plasmin, tissue plasminogen activator (tPA), plasminogen activator inhibitor (PAI-1), a few MMPs, and tissue inhibitors of matrix metalloproteinases (TIMPs) (*24*).

Among MMPs, MMP-9 has emerged as a key player in synaptic plasticity (*25*–*31*). It is essential for the maintenance of LTP (*25*), as well as for structural plasticity, promoting a sustained increase of dendritic spines volume evoked by neuronal stimulation (*32*). MMP-9 is produced as a zymogen in its pro-form and is released upon increase in neuronal activity to the extracellular space, where it acts proteolytically. This enzymatic activity is tightly regulated. Pro-MMP-9 can be pre-processed by plasmin, and the active MMP-9 is inhibited by TIMP1 to limit the proteolysis (*24*, *25*, *33*–*35*). Thus, BDNF and MMP-9 may undergo extracellular interplay with each other, including potential proBDNF to mBDNF processing by MMP-9 (*19*–*22*).

Both BDNF and MMP-9 are essential in synaptic plasticity–dependent phenomena, such as learning and memory. Additionally, they are engaged in neuropsychiatric conditions caused by aberrant synaptic plasticity, such as schizophrenia, addiction, depression, autism spectrum disorders, or epilepsy (*6*, *36*, *37*). However, the timing and location of MMP-9 action and interaction with BDNF signaling at individual synapses remain elusive. BDNF has been shown to be rapidly secreted (within seconds) upon cell depolarization (*38*), theta-burst stimulation (*39*), or structural LTP (sLTP)–inducing glutamate uncaging (*40*). TrkB activation also occurs rapidly, reaching the maximum within 1 min from the onset of stimulation (*40*). On the other hand, MMP-9 has been mostly implicated in the plasticity lasting over a couple of dozen minutes (*25*, *32*). Similarly, studies show that MMP-9 release and enzymatic activity occur several minutes after synaptic stimulation (*33*, *34*, *41*). Therefore, the question remains whether MMP-9 activity may be involved in maturation of proBDNF, and whether it leads to TrkB activation as both proteins need to meet at the same synapse at the same time (*20*–*22*).

Here, we investigated the spatiotemporal dynamics of MMP-9 and BDNF release upon activation. Using total internal reflection fluorescence (TIRF) microscopy, we observed a release of vesicles containing MMP-9 and those with BDNF to find that they are released in a similar time window. Furthermore, to pinpoint the dynamic processes of structural plasticity, we used glutamate uncaging to show that MMP-9 proteolytic activity increases exclusively around stimulated dendritic spines. Next, we used two-photon fluorescence lifetime imaging microscopy (2pFLIM) to image a Förster resonance energy transfer (FRET) sensor of TrkB activation on individual dendritic spines upon glutamate uncaging (*40*, *42*–*44*). By blocking MMP-9 activity, we have shown its essentiality for structural plasticity as well as for TrkB activation. Moreover, in a cell-free assay, we have confirmed MMP-9–driven direct cleavage of proBDNF to mBDNF.

Overall, our findings support the hypothesis of the cooperative role of MMP-9 and BDNF in synaptic plasticity. Both proteins are released from a stimulated spine, where MMP-9 proteolytic activity increases. That leads to autocrine TrkB activation and structural plasticity. This precisely described mechanistic link between MMP-9, BDNF, and synaptic plasticity provides important insights into brain function in both health and disease, with potential relevance for understanding neuropsychiatric disorders related to dysfunctional synapses.

## RESULTS

### MMP-9 and BDNF are stored and released from dendritic spines

BDNF has been previously shown to be postsynaptically released from a dendritic spine upon stimulation to activate its receptor, TrkB. The TrkB activation was rapid, with its onset shorter than a minute, and sustained, lasting over 20 min (*40*). On the other hand, MMP-9 has typically been implicated in synaptic plasticity at later stages, typically 10 min or more post-stimulation (*25*, *32*, *45*). However, its release and role in the earlier phases of synaptic plasticity have not been extensively studied.

**Fig. 1.**
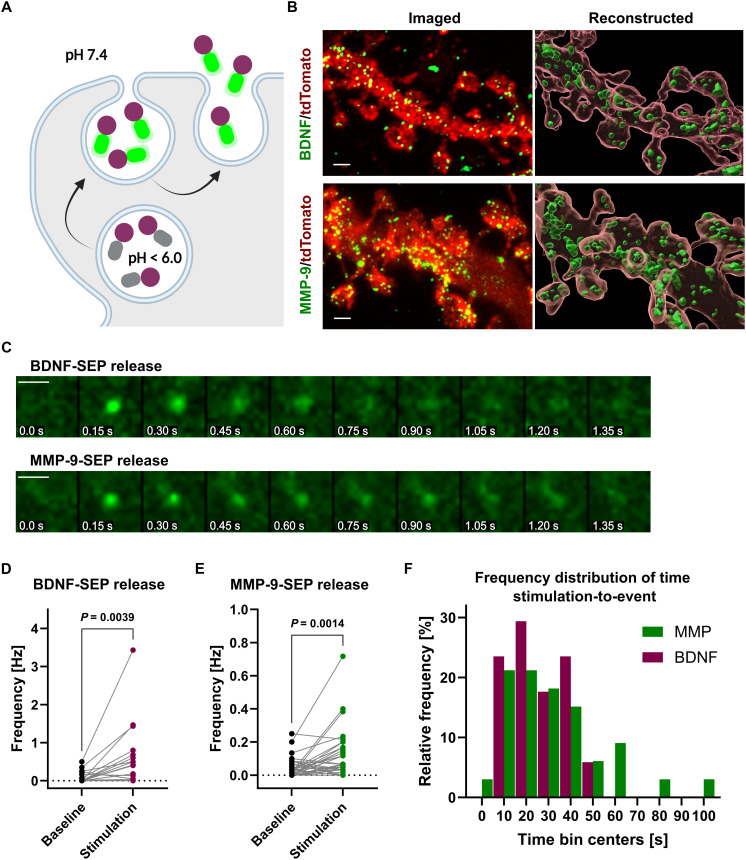
MMP-9 and BDNF release during synaptic plasticity. (**A**) Scheme of SEP fluorescence changes during vesicle release. SEP fluorescence is quenched by low (<6.0) pH inside vesicles. Stimulation promotes exocytosis, and once the vesicle fuses with the cell membrane, pore opens and the acidic environment in the vesicle’s lumen is neutralized (pH ~ 7.4), allowing for observation of fluorescence of SEP. Created in BioRender. Kalita, K. (2025) https://BioRender.com/6yuj77a. (**B**) Expansion microscopy immunofluorescence staining showing vesicles containing BDNF-SEP (top) and MMP-9–SEP (bottom). Merged photos of SEP signal and tdTomato fluorescence coming from the neuronal dendrite (left-hand side panels) of physically expanded neurons. Results of the graphical reconstruction of vesicles and cell morphology (right-hand side panels). Scale bar, 2 μm. (**C**) Exemplary time lapses of BDNF-SEP and MMP-9–SEP vesicle releases. Scale bar, 2 μm. (**D**) Graph representing the frequency of BDNF-SEP vesicle release under basal conditions and during electrical stimulation using the LTP protocol. Dots represents frequency of events paired for individual recordings (*n* = 20 recordings, 17 cells). Paired *t* test (*P* = 0.0039). (**E**) Same as (D) but for MMP-9–SEP vesicle release (*n* = 46 recordings, 44 cells). Paired *t* test (*P* = 0.0014). (**F**) Histogram showing the frequency distribution of times of occurrence of successfully evoked BDNF-SEP (magenta) and MMP-9–SEP (green) events during the stimulation with LTP protocol lasting from time 0 to 60 s. Times of release were binned into 10-s intervals.

Therefore, we aimed to analyze whether MMP-9 stimuli-evoked release occurs with a similar dynamic to BDNF. First, we examined whether BDNF and MMP-9 are stored in dendritic spines, positioning them for rapid engagement in synaptic plasticity. To achieve this, we created a fusion protein by attaching pH-sensitive green fluorescent protein (GFP) [superecliptic pHluorin (SEP); Fig. 1A; (*46*)] to a C terminus of MMP-9. For comparison with BDNF, we used the previously described BDNF-SEP construct (*40*). We transfected rat hippocampal neuronal cell cultures with plasmids encoding tdTomato (for visualizing dendritic spine morphology), together with plasmids for either MMP-9–SEP or BDNF-SEP. We have used immunostaining to image MMP-9– and BDNF-containing vesicles. To improve the final resolution of the imaged neurons, we used the expansion microscopy technique [expanded fourfold, as measured by nuclei signal; fig. S1, A and B; (*47*)]. We then imaged these samples using confocal microscopy, and images of dendritic spines were used for the reconstruction of vesicles and cell morphology using Imaris Image Analysis Software (Fig. 1B). We confirmed the storage of both BDNF- and MMP-9–containing vesicles within dendritic spines. BDNF-containing vesicles had an average diameter of 149.6 ± 2.4 nm, whereas those containing MMP-9 had an average diameter of 135.9 ± 2.6 nm (fig. S1C).

Having confirmed the proximity of these vesicles to dendritic spines, we next investigated the timeline of their release following increased neuronal activity. Using TIRF microscopy (Fig. 1C) and electrical stimulation of neurons in an LTP-like protocol (30 bursts in total, each consisting of twenty 1-ms-long pulses, delivered every 2 s), we demonstrated the rapid release of both MMP-9 and BDNF vesicles upon stimulation (Fig. 1, C to F, and movies S1 and S2), observed as increased frequency of release events. Most vesicles were released rapidly within 50 s after the onset of stimulation (Fig. 1F). The rise time and decay time of release events were different between BDNF- and MMP-9–containing vesicles, namely, both rise time and decay time of the release were somewhat delayed in the case of MMP-9, when compared with BDNF (fig. S2).

### Glutamate uncaging increases MMP-9 enzymatic activity at a stimulated spine

The aforementioned experiments have demonstrated that MMP-9 protein is rapidly released upon electrical stimulation of the hippocampal neurons in dissociated culture in a manner similar to BDNF. However, electrical stimulation activates multiple cellular inputs simultaneously and does not fully replicate physiological conditions where only a few synaptic contacts are typically engaged. Additionally, to fulfill its role at a synapse, MMP-9 needs to be enzymatically active upon its release. Therefore, we used glutamate uncaging, which allows for precise, single-spine resolution stimulation, and checked whether it leads to an increase in MMP-9 proteolytic activity.

**Fig. 2.**
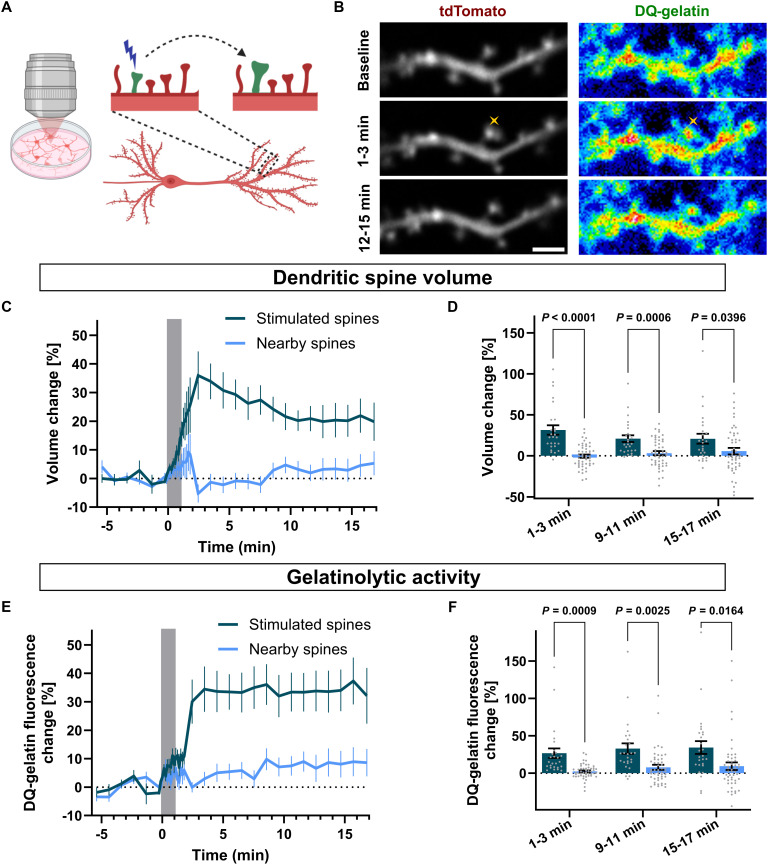
Proteolytic activity of MMP-9 during sLTP. (**A**) Experimental arrangement: tdTomato-expressing neurons from hippocampal cell culture were imaged under two-photon microscope. Individual dendritic spine was stimulated using sLTP-inducing uncaging protocol. Created in BioRender. Kalita, K. (2025) https://BioRender.com/vum10zr. (**B**) Time-lapse t-integrated (t-stack) images of spines stimulated by uncaging protocol and imaged in two separate channels for tdTomato fluorescence and of DQ-gelatin signal. Images show average of frames collected during baseline, at 1 to 3 min after uncaging, and at 12 to 15 min after uncaging. Yellow crosses indicate a spot of two-photon uncaging. Scale bar, 2 μm. (**C**) Averaged time courses of changes in tdTomato fluorescence (measured as Δ*F*/*F*_0_). Data are means ± SEM. Gray box indicates timing of sLTP-inducing protocol. (**D**) Statistical analysis of spine volume change in (C). Averaged spine volume changes for three phases: 1 to 3 min (transient), 9 to 11 min, and 12 to 15 min (sustained). Gray dots represent individual values for measurements. Bars, means ± SEM. Stimulated spines (dark blue; *n* = 26 spines, 15 cells), nearby spines (pale blue; *n* = 49 spines, 15 cells). Repeated-measures ANOVA: Time (*P* = 0.4584); Stimulation (*P* < 0.0001); Time × Stimulation (*P* = 0.0245), followed by Tukey’s multiple comparisons test (*P* values indicated on the graph). (**E**) Averaged time courses of changes in DQ-gelatin fluorescence (measured as Δ*F*/*F*_0_). All markings as in (C). (**F**) Statistical analysis of DQ-gelatin fluorescence change in (E). All markings as in (D). Repeated-measures ANOVA: Time (*P* = 0.0483); Stimulation (*P* = 0.0002); Time × Stimulation (*P* = 0.9774), followed by Tukey’s multiple comparisons test (*P* values indicated on the graph).

To assess the local availability of the active MMP-9 near stimulated synapses, we measured its enzymatic (gelatinolytic) activity. We transfected rat neuronal cultures with tdTomato to visualize spine morphology. On the day of the experiment, neurons were incubated in a buffer containing 4-methoxy-7-nitroindolinyl (MNI)–caged-l-glutamate and DQ-gelatin, a fluorogenic gelatin, which is a canonical substrate for MMP-9. DQ-gelatin consists of highly quenched, fluorescein-labeled gelatin, which upon proteolytic digestion emits green fluorescence, indicating local enzymatic activity of MMP-9 [Fig. 2, A and B, and movie S3; (*48*)]. We stimulated single dendritic spines of tdTomato-expressing neurons by glutamate uncaging using sLTP-inducing protocol (*40*). We measured the structural plasticity in the tdTomato (red) fluorescence channel (Fig. 2C) and DQ-gelatin digestion in the green channel (Fig. 2E).

Glutamate uncaging triggered a rapid increase in tdTomato fluorescence at stimulated spines, indicating their enlargement (Fig. 2, C and D). Additionally, the stimulation protocol proved its specificity, as unstimulated, neighboring spines remained unchanged. The sLTP protocol also caused a rapid increase in the DQ-gelatin signal (increase in green fluorescence) at the stimulated spine (Fig. 2, E and F). The gelatinolytic activity was not elevated over unstimulated neighboring spines, which shows that MMP-9 acts locally after the release.

### MMP-9 is rapidly released during sLTP in an NMDAR-dependent manner

**Fig. 3.**
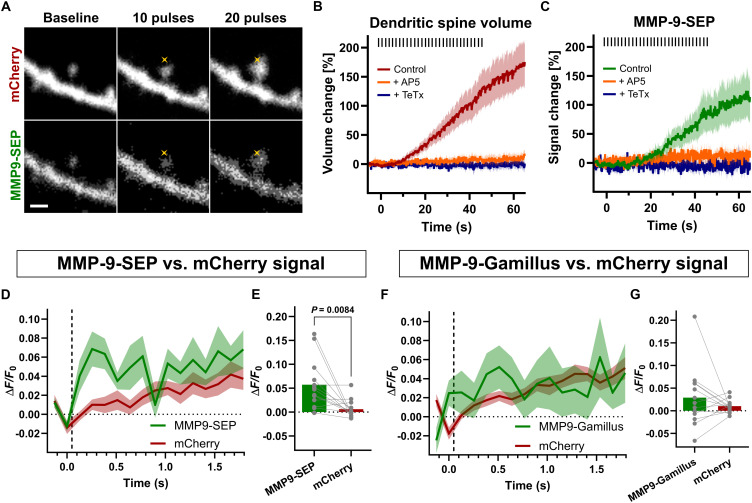
MMP-9 release during sLTP. (**A**) Time-lapse t-integrated (t-stack) images of spines stimulated by uncaging protocol—average of 10 consecutive frames collected after indicated uncaging pulses. Separate channels of MMP-9–SEP and mCherry are shown. Crosses indicate a spot of two-photon uncaging. Scale bar, 1 μm. (**B**) Averaged time courses of changes in fluorescence of mCherry measured as Δ*F*/*F*_0_ for spines stimulated by uncaging. Orange and blue traces represent data in AP5 and TeTx presence, respectively. Data are means ± SEM. Timing of glutamate uncaging laser pulses during sLTP protocol are indicated by black bars. Control (*n* = 13 spines, 3 cells); + AP5 (*n* = 13 spines, 3 cells); + TeTx (*n* = 12 spines, 1 cell). (**C**) Same as in (B) but for corresponding MMP-9–SEP fluorescence. (**D**) Averaged time courses of changes in MMP-9–SEP and corresponding mCherry fluorescence (measured as Δ*F*/*F*_0_) for stimulated spines measured between sweeps corresponding to 2nd and 18th uncaging pulse. Data are means ± SEM. Timing of glutamate uncaging laser pulses (between imaging frames) during sLTP protocol is indicated by dashed vertical line. (**E**) Peak of the uncaging-triggered change (calculated as a mean of Δ*F*/*F*_0_ for the three subsequent frames after uncaging pulse: 0.128 to 0.384 s). Dots with lines represent values for individual spines for MMP9-SEP and mCherry fluorescence paired together. Bars are means. Paired *t* test (*P* = 0.0084). (**F**) Same as in (D) but for MMP-9–Gamillus and mCherry fluorescence (*n* = 13 spines, 5 cells). (**G**) Same as in (E) but for MMP-9–Gamillus and mCherry fluorescence. Paired *t* test (*P* = 0.3262).

Our data using TIRF microscopy and electrical stimulation of dissociated hippocampal cultures indicate that MMP-9 can be released in a time similar to BDNF. However, our electrical stimulation protocol leads to simultaneous activation of multiple cell inputs. Therefore, to study the MMP-9 release on a single spine/synapse, we used glutamate uncaging–induced sLTP protocol applied to mouse hippocampal organotypic cultures (*49*–*51*). We cotransfected the cultures with MMP-9–SEP and mCherry and focused on dendritic spines of CA1 pyramidal neurons. Glutamate uncaging caused a rapid spine enlargement (Fig. 3, A and B), accompanied by increased MMP-9–SEP fluorescence in the spine (Fig. 3, A and C, and movie S4). Similar to the results from electrical stimulation, glutamate uncaging–driven MMP-9 release occurred within seconds of the stimulation onset.

Previous studies have shown that structural plasticity (*3*, *52*) and BDNF release (*40*) both depend on NMDAR (*N*-methyl-d-aspartate receptor) activation. To determine whether MMP-9 release is also NMDAR dependent, we repeated the uncaging protocol in the presence of an NMDAR antagonist, AP5. This treatment abolished the SEP fluorescence increases during stimulation and, consistent with earlier studies, blocked spine enlargement (Fig. 3, B and C). Moreover, postsynaptic coexpression of tetanus toxin light chain (TeTx), which blocks the synaptobrevin-dependent exocytosis (*40*, *53*), abolished both SEP fluorescence increases and spine volume change during uncaging (Fig. 3, B and C). These results indicate that MMP-9 release depends on exocytosis and NMDAR activation.

However, the changes in MMP-9–SEP signal are the combination of SEP fluorescence changes associated with the MMP-9 release and the increase in the number of molecules associated with dendritic spine enlargements. Therefore, to better visualize the rapid release of MMP-9 upon uncaging pulses, which directly stimulate the spine, we analyzed fluorescence signals of MMP-9–SEP and mCherry between uncaging pulses. Both signals were divided into 2-s sweeps, which represent a single uncaging trial during sLTP protocol. First, seven sweeps recorded without uncaging were followed by uncaging sweeps, where the uncaging pulse was always between the second and the third frame in the sweep (fig. S3, A and B). Next, we averaged the relative MMP-9–SEP fluorescence changes for the 2nd to 18th uncaging pulses (sweeps 9 to 25), where the most pronounced changes occur, and plotted over time alongside mCherry fluorescence (Fig. 3, D and E). While mCherry fluorescence showed a slow increase, reflecting spine volume changes, MMP-9–SEP fluorescence had a faster onset, consistent with the rapid MMP-9 release in response to each uncaging pulse.

The observed accumulation of the MMP-9–SEP signal can be explained not only by MMP-9 being released to the extracellular space (increase in SEP fluorescence due to pH change) but also by the accumulation of MMP-9–SEP in the enlarging dendritic spine. To confirm that MMP-9–SEP fluorescence increases were mainly due to pH changes associated with vesicular MMP-9 release rather than an increase in the number of vesicles in the spine, we performed similar experiments using Gamillus (pH-stable GFP variant), fused to MMP-9 (*54*). The Gamillus signal should not change upon MMP-9–Gamillus release, in contrast to MMP-9–SEP. However, due to spine enlargement, it showed a signal change similar to that of MMP-9–SEP signal throughout the entire uncaging procedure (fig. S4). Therefore, similarly as in the MMP-9–SEP experiment, we analyzed the signals from MMP-9–Gamillus and mCherry within 2-s sweeps between uncaging pulses (fig. S3, C and D). Next, we averaged the relative MMP-9–Gamillus and mCherry fluorescence changes for the 2nd to 18th uncaging pulses (sweeps 9 to 25). In contrast to MMP-9–SEP, we have not observed a statistically significant difference between MMP-9–Gamillus and mCherry fluorescence changes (Fig. 3, F and G). Therefore, these results further support the hypothesis that MMP-9 is rapidly released at synapses undergoing sLTP.

### MMP-9 activity is required for structural plasticity

So far, our experiments have shown that single-spine sLTP protocol causes a rapid release of MMP-9, which coincides with an increase in MMP-9 enzymatic activity around the stimulated spine. We have also observed that blocking structural plasticity can be achieved by either inhibiting NMDARs or blocking exocytosis from a postsynaptic neuron. Next, we sought to examine whether blocking MMP-9 activity affects structural plasticity, starting from the early stages of sLTP.

**Fig. 4.**
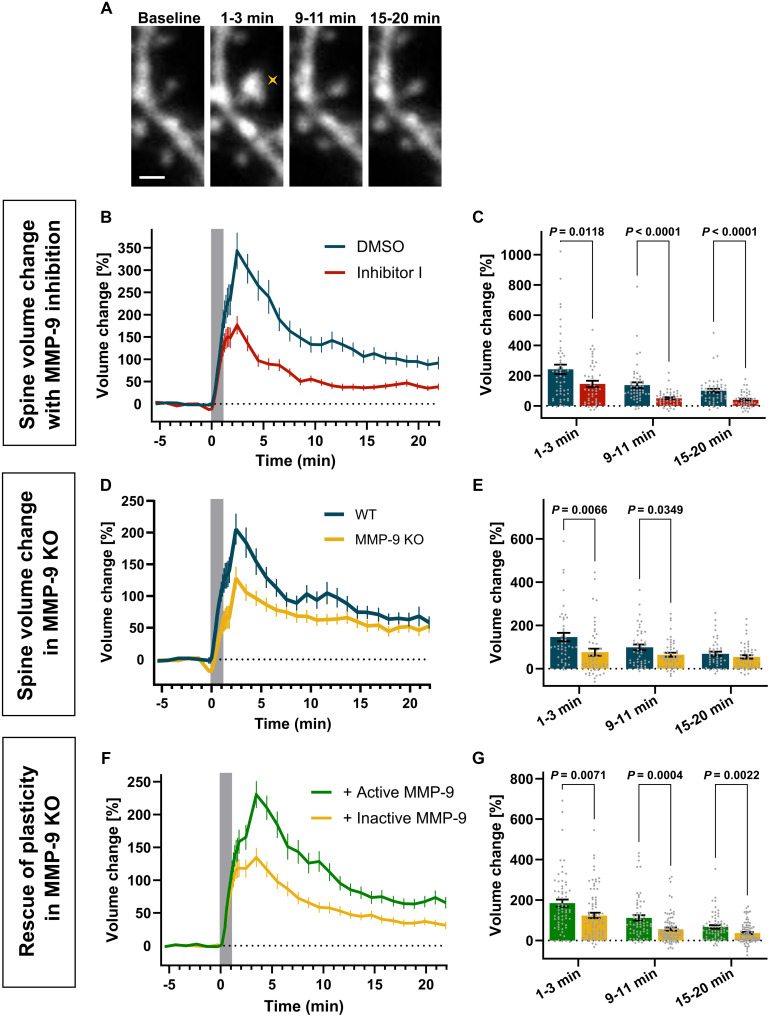
Spine-head enlargement during sLTP depends on MMP-9 activity. (**A**) Time-lapse images showing dendritic spines stimulated at 0 min by glutamate uncaging (yellow cross). Scale bar, 1 μm. (**B**) Averaged spine volume changes following uncaging in the presence DMSO or Inhibitor I. Data are means ± SEM. Uncaging period indicated by gray box. (**C**) Statistical analysis of (B). Averaged spine volume changes for three phases: 1 to 3 min, (transient), 9 to 11 min, and 15 to 20 min (sustained). Gray dots, individual spines; bars, means ± SEM. DMSO (blue; *n* = 50 spines, 25 cells, 15 animals), Inhibitor I (red; *n* = 46 spines, 21cells, 10 animals). Repeated-measures ANOVA: Time (*P* < 0.0001); Inhibitor (*P* < 0.0001); Time × Inhibitor (*P* = 0.1534), followed by Tukey’s multiple comparisons test (*P* values indicated on the graph). (**D**) Averaged spine volume changes following uncaging in slices from WT and MMP-9 KO mice. All markings as in (B). (**E**) Statistical analysis of (D). WT (blue; *n* = 44 spines, 12 cells, 7 animals) and MMP-9 KO animals (yellow; *n* = 51 spines, 14 cells, 7 animals). Repeated-measures ANOVA: Time (*P* < 0.0001); MMP-9 (*P* = 0.0070); Time × MMP-9 (*P* = 0.0669), followed by Tukey’s multiple comparisons test (*P* values indicated on the graph). (**F**) Averaged spine volume changes following uncaging in slices from MMP-9 KO mice overexpressing either active MMP-9 or inactive MMP-9. All markings as in (B). (**G**) Statistical analysis of (F). Active MMP-9 (green; *n* = 64 spines; 17 cells, 7 animals) and inactive MMP-9 (yellow; *n* = 82 spines, 21 cells, 7 animals). Repeated-measures ANOVA: Time (*P* < 0.0001); MMP-9 (*P* < 0.0001); Time × MMP-9 (*P* = 0.0429), followed by Tukey’s multiple comparisons test (*P* values indicated on the graph).

To test this hypothesis, we transfected neurons in organotypic culture with a GFP plasmid and imaged transfected neurons in the CA1 field using two-photon microscopy. Next, we measured changes in spine volume as a change in GFP fluorescence for individual spines (*40*, *52*, *55*). To block MMP-9, we incubated organotypic slices in the presence of either MMP Inhibitor I or dimethyl sulfoxide (DMSO) for control. In control conditions, we observed that glutamate uncaging resulted in a rapid spine volume increase, which subsequently decreased but stabilized at an elevated level over the baseline size. This sustained phase began approximately 8 min after stimulation (Fig. 4, A to C, and movie S5). In comparison, dendritic spines imaged in the presence of Inhibitor I displayed a markedly lower increase following the stimulation.

For further analysis, we assessed dendritic spine volume change at three time windows: the transient phase (1 to 3 min post-uncaging) and the sustained phase (9 to 11 min and 15 to 20 min post-uncaging). The presence of Inhibitor I significantly reduced the spine volume increase across all phases (Fig. 4, B and C). This result indicated that the activity of MMPs, including MMP-9, is crucial for both the induction and maintenance of spine enlargement.

There are over 20 MMPs with overlapping substrate specificity (*56*, *57*), and none of the commercially available inhibitors are specific for MMP-9. To exclude potential unspecific effects of Inhibitor I on other proteases, we used organotypic hippocampal slice cultures from MMP-9 knockout (MMP-9 KO) mice and their wild-type (WT) littermates (Fig. 4, D and E) for further experiments. MMP-9 KO significantly reduced spine volume changes during the transient phase of plasticity (1 to 3 min; Fig. 4E) and early, sustained phase (9 to 11 min), but not during the later sustained phase (15 to 20 min). Furthermore, we aimed to determine whether the decreased sLTP phenotype observed in MMP-9 KO mice could be rescued by overexpression of MMP-9. To test this, we cotransfected hippocampal organotypic slices from MMP-9 KO mice with the GFP-coding plasmid, and a plasmid encoding either full-length MMP-9 (active) or its mutant, E402A [inactive; Fig. 4, F and G; (*58*)]. Overexpression of active MMP-9 significantly enhanced plasticity across all phases of sLTP, with spine volume increases observed during both transient and sustained phases (Fig. 4, F and G).

Together, these findings suggest that MMP-9 acts rapidly during synaptic plasticity and is essential for the induction of LTP as well as for triggering biochemical processes that lead to sustained spine enlargement.

### TrkB activation depends on MMP-9 activity

Our experiments described above demonstrated that MMP-9 was essential not only during plasticity maintenance but also for the early phases of sLTP. Other studies showed that MMP-9 could potentially convert proBDNF into its mature form, mBDNF (*20*–*22*, *59*, *60*). Given that BDNF is a key protein involved in early sLTP (*40*) and our experiments showed that both MMP-9 and BDNF had similar release timelines following synaptic stimulation, we hypothesized that MMP-9 might influence synaptic plasticity by promoting BDNF maturation and, thus, TrkB activation.

**Fig. 5.**
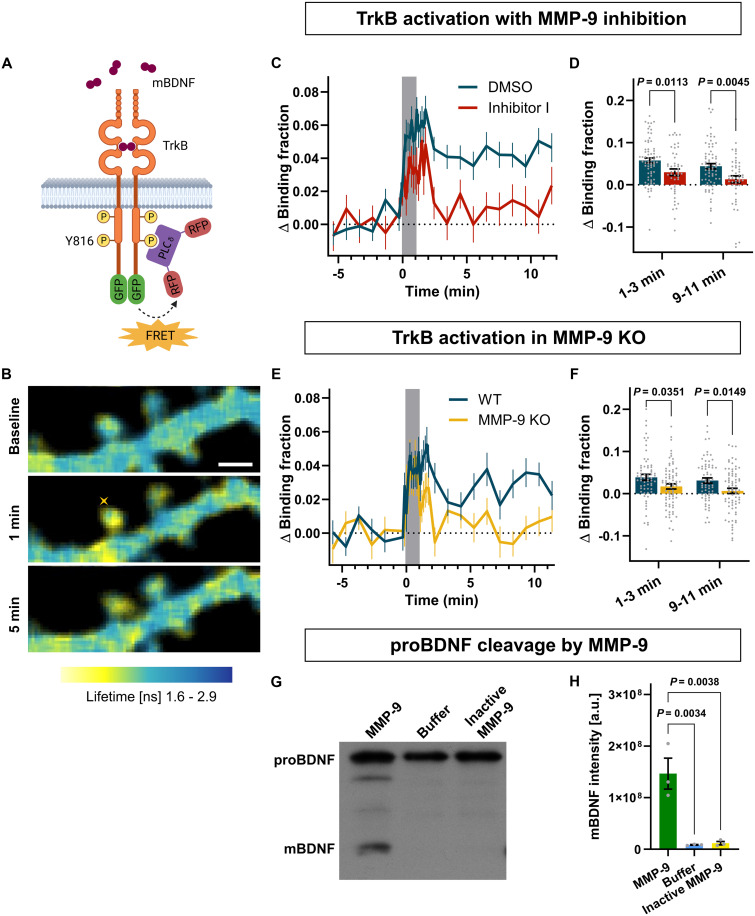
TrkB activation depends on MMP-9 activity. (**A**) Scheme of the TrkB FRET sensor. Created in BioRender. Kalita, K. (2025) https://BioRender.com/qaducze. (**B**) Example FLIM images showing TrkB activation. Warmer colors represent higher TrkB activity. Yellow cross indicates uncaging spot. Scale bar, 1 μm. (**C**) Averaged TrkB activation changes (Δ binding fraction) in dendritic spines following uncaging in the presence of DMSO or Inhibitor I. Data are means ± SEM. Gray box indicates uncaging period. (**D**) Statistical analysis of (C). Averaged TrkB activation in stimulated spines during transient (1 to 3 min) and sustained phase (9 to 11 min). Gray dots, individual spines; bars, means ± SEM. DMSO (blue; *n* = 70 spines, 27 cells, 16 animals) and Inhibitor I (red; *n* = 49 spines, 21 cells, 10 animals). Repeated-measures ANOVA: Time (*P* = 0.0010); Inhibitor (*P* = 0.0013); Time × Inhibitor (*P* = 0.7403), followed by Šídák’s multiple comparisons test (*P* values indicated on the graph). (**E**) Averaged TrkB activation changes in dendritic spines following uncaging in WT or MMP-9 KO slices. All markings as in (C). (**F**) Statistical analysis of (E). All markings as in (D). WT (blue, *n* = 66 spines; 22 cells, 10 animals), MMP-9 KO (yellow, *n* = 73 spines; 25 cells, 11 animals). Repeated-measures ANOVA: Time (*P* = 0.0257); MMP-9 KO (*P* = 0.0054); Time × MMP-9 KO (*P* = 0.7238), followed by Šidák’s multiple comparison test (*P* values indicated on the graph). (**G**) Example immunoblot of digestion reaction of proBDNF incubated with either active MMP-9, inactive MMP-9 (E402A), or the reaction buffer. Bands correspond to proBDNF (~26 kDa) and mBDNF (~14 kDa). (**H**) Quantification of immunoblots of three digestion reactions. Gray dots, individual values of mBDNF band intensity in separate experiments; bars, mean ± SEM. One-way ANOVA (*P* = 0.0021) followed by Tukey’s multiple comparisons test (*P* values indicated on the graph).

To test this hypothesis, we transfected neurons in hippocampal organotypic cultures with a FRET sensor of TrkB, the primary receptor for mBDNF. The sensor is composed of two fusion proteins: TrkB linked with GFP (donor) and SH2 domain of phospholipase C-γ (PLC-γ) linked with a pair of mRFP1 proteins (acceptor). Upon BDNF binding, TrkB autophosphorylates its cytoplasmic domain, allowing for SH2-mRFP1 domain interaction and effective FRET to occur (Fig. 5A) (*40*). Glutamate uncaging decreased the fluorescence lifetime of GFP, which reflected an increase in the binding fraction between TrkB and SH2 domain of the sensor (Fig. 5B and movie S6), and thus was indicative of TrkB activation. Using the sensor, we measured TrkB activation during uncaging-induced sLTP in the presence of either Inhibitor I or DMSO for control (Fig. 5, C and D). In the presence of Inhibitor I, TrkB activation was significantly reduced in both the transient and sustained phases compared to the DMSO control (Fig. 5D), showing that MMP-9 activity was necessary for TrkB activation.

To confirm MMP-9–specific role, we used hippocampal organotypic cultures from MMP-9 KO mice and their WT littermates (Fig. 5E) and measured TrkB activation using the FRET sensor. Similar to the effects of Inhibitor I, TrkB activation was significantly attenuated in MMP-9 KO mice during both transient and sustained phases of plasticity (Fig. 5, E and F). These results confirm that MMP-9 plays an important role in activating TrkB following repeated glutamate uncaging, which induces structural changes in dendritic spines.

Finally, to determine whether MMP-9 can directly cleave proBDNF into mBDNF, we conducted a cell-free assay where we incubated recombinant proBDNF with recombinant active MMP-9 overnight at 37°C in a reaction buffer. As controls, we used recombinant inactive mutant (E402A) of MMP-9 or buffer alone (*58*). The cleavage of proBDNF was quantified by Western blot, using anti-BDNF antibody recognizing both (pro-form and mature form). Analysis showed that incubation with active MMP-9, but not with inactive MMP-9, causes cleavage of proBDNF (Fig. 5, G and H). The incubation produces not only a band corresponding to mBDNF but also weaker bands with higher molecular weight. In conclusion, these results indicate that MMP-9 may directly cleave proBDNF into mBDNF, leading to TrkB activation during LTP.

## DISCUSSION

Here, we demonstrate that (i) BDNF and MMP-9 are both rapidly, within seconds, released from the dendritic spines and (ii) MMP-9 is needed for transient and sustained phases of structural synaptic plasticity. Furthermore, (iii) MMP-9 cleaves proBDNF to produce its mature form and (iv) MMP-9 inhibition impairs the activation of BDNF receptor TrkB. These results demonstrate a mechanistic link between MMP-9 and BDNF in controlling synaptic plasticity.

### MMP-9 and BDNF are rapidly, within seconds, released from the dendritic spines

Our results have shown that vesicles containing MMP-9, similar to those with BDNF, are stored at dendritic spines. BDNF is widely considered to be stored and released from large dense-core vesicles (*11*, *61*), and the vesicle size measured in our expansion microscopy experiment is within the range for this type of vesicles. MMP-9 has been previously shown to be present in 160- to 200-nm vesicles in N2a neuronal cells (*62*) and in dense-core vesicles in cancer cells (*63*). Considering the vesicle size, our study suggests MMP-9 release from dense-core vesicles in neurons. Incidentally, previous reports showed an expression of MMP-9 protein within the dendritic spines, predominantly in small ones, which are more prone to plastic changes (*33*, *52*, *64*, *65*).

Synaptic localization of BDNF and MMP-9 suggests that these proteins are readily releasable. While the rapid release of BDNF has been shown before (*40*), we have demonstrated the rapid release of MMP-9 upon stimulation using two different methods: TIRF microscopy combined with field electrical stimulation and two-photon imaging with two-photon glutamate uncaging. The first method provides excellent temporal resolution, enabling us to visualize individual release events (Fig. 1), while two-photon imaging combined with glutamate uncaging allowed us to observe MMP-9 release on a single dendritic spine (Fig. 3). The results presented herein show that MMP-9 release occurs as early as 2 to 3 s after the first uncaging pulse. The release is sustained by repeatable stimulation of sLTP protocol for up to 40 s, and further uncaging fails to cause MMP-9 exocytosis, which may be due to the depletion of spine vesicles. Notably, the timescale of the MMP-9 release is thus similar to that of BDNF release (*40*). Additionally, because of the single-spine specificity of glutamate uncaging, we have shown that upon release, MMP-9 is enzymatically active already within 2 min from activation (Fig. 2) and that the protease does not diffuse significantly from the stimulated spine. This is probably caused by the size of the protease (92 kDa) and interactions with the ECM through the MMP-9 hemopexin-like domain and fibronectin type 2 domains (*66*–*68*).

Previously published data indicated longer release times (5 min), although the earlier time points were not apparently investigated (*33*). Additionally, the earliest time point demonstrating extracellular enzymatic activity of MMP-9 was 10 min after stimulation, shown either by cleavage of its substrate, β-dystroglycan (*33*), or 15 min after stimulation, indicated by increased fluorescence of DQ-gelatin (*48*). This discrepancy is probably caused by the low sensitivity and temporal resolution of previously used methods. Michaluk *et al.* (*33*) collected the medium from stimulated culture and analyzed the presence of MMP-9 with either gel zymography or cleavage of β-dystroglycan in cell lysates, while Lepeta *et al.* (*48*) used chemical LTP to induce synaptic changes and the gelatinolytic activity of MMP-9. Moreover, it was previously shown that MMP-9 was locally translated in response to synaptic stimulation and then released to contribute to synaptic plasticity, the processes that require minutes to occur (*69*). Yet, our and other data (*33*, *62*, *64*, *65*) show that there is a readily releasable pool of MMP-9. Notably, the spine volume increased before a significant increase in gelatinolytic activity (Fig. 2, C and E). This effect is most probably caused by the time needed for the accumulation of fluorescent gelatin to be distinguishable from baseline fluorescence. This was previously shown in an enzymatic assay (*58*), where enzyme concentrations are much larger than those present at a single spine. Moreover, it can be observed that the signal from the DQ-gelatin does not drop after initial increase (Fig. 2, C and E), in contrast to the spine volume, showing that DQ-gelatin digestion is irreversible. Therefore, both the quick release of MMP-9 and the increase in its extracellular proteolytic activity suggest that the enzyme can be responsible for early phases of LTP through proteolytic cleavage of its synaptic substrates, including proBDNF. It is possible, however, that an initial increase in spine volume and TrkB activation is regulated by released mBDNF, which was pre-processed intracellularly.

### MMP-9 is needed for transient and sustained phases of structural synaptic plasticity

Our observations show that MMP-9 is not only rapidly released and enzymatically active outside of the cell but also essential for the transient and sustained phase of sLTP (Fig. 4). This is an unexpected observation, positioning MMP-9 among early executors of the synaptic plasticity cascade. The involvement of MMP-9 in the early phase of sLTP is in contrast with previous studies, showing its involvement mainly in the late phase of LTP (*25*, *28*, *32*, *45*). However, structural plasticity does not necessarily follow the same signaling pathways as those leading to synaptic strengthening and electrophysiological LTP. A recent study by Stein *et al.* (*70*) dissociated the NMDAR-dependent structural and functional LTP by pointing to the role of non-ionotropic NMDAR signaling through p38 mitogen-activated protein kinase (MAPK). Additionally, differences in LTP-evoking protocols might explain why the MMP-9 dependence of the transient phase of sLTP has not been observed before. Wang and coworkers (*32*) have shown that MMP-9 is both necessary and sufficient to drive spine enlargement and synaptic potentiation concomitantly by using theta-stimulation–induced LTP paired with a depolarization of postsynaptic neurons. This protocol, due to backpropagating action potential evoked by depolarization, could itself lead to the postsynaptic exocytotic release of BDNF and MMP-9, even without presynaptic glutamate release. The protocol used by Wang and coworkers (*32*) induces initial spine enlargement even in the presence of postsynaptic botulinum toxin, which blocks exocytosis (*71*). In contrast, in our experiments, the presence of postsynaptic exocytosis inhibitor (TeTx) blocked both sLTP and MMP-9 release (Fig. 3B).

Some studies reported BDNF involvement in both early- and late-phase LTP, while others found its effects restricted to late-phase LTP (*40*, *72*–*74*). An important factor contributing to these discrepancies might be the type of stimulation protocol used in different studies. Chen *et al.* (*75*) demonstrated protocol-dependent mechanisms underlying early-phase LTP, showing that distinct induction protocols use different molecular signaling. The study revealed that tetanic stimulation elicited LTP independent of TrkB signaling, whereas 3× theta burst stimulation in the presence of a TrkB blocker impaired the late phase but not the early phase of LTP. These findings strongly suggest that the molecular mechanisms underlying early-phase LTP induction are highly protocol dependent.

In our results, Inhibitor I affected sLTP at the sustained phase, which agrees with previously published electrophysiology and imaging data (*25*, *28*, *32*, *76*). However, MMP-9 gene deletion did not cause sLTP inhibition at 15 to 20 min following sLTP induction. Noteworthy, such discrepancies in genetic and chemical perturbation of MMP-9 on plasticity were also described previously. In Nagy *et al.* (*25*), LTP in MMP-9 KO animals was impaired in both early- and late-phase LTP, but the use of chemical inhibitors of MMP-9 affected only late-phase LTP. It is possible that MMP-9 KO mice exhibit compensatory mechanisms, for example, by other MMPs. Additionally, although MMP-9 KO mice are kept on C57BL/6 background and the line is maintained by breeding heterozygotes together, WT littermates, which have been used in the study, have markedly lower increase of spine volume in comparison to slices prepared from WT C57BL/6J mice (Fig. 4). Therefore, the difference in sLTP phenotypes may be explained by some unaccounted genetic differences between the two mouse lines. It has been recently shown that even closely related mice substrains C57BL/6J and C57BL/6N express substantial metabolic and genetic differences (*77*). Additionally, differences in the magnitude of volume change during structural plasticity between different transgenic animals have also been previously observed (*40*). Yet, the rescue experiment where we express MMP-9 in the slices obtained from MMP-9 KO animals (Fig. 4, F and G) shows that MMP-9 enzymatic activity is important in all phases of sLTP. It is also possible that, due to the existence of redundant signaling pathways, we might observe a mixture of different effects when inhibiting MMP-9 activity. For example, it has been previously shown that MMP-9 can influence synaptic plasticity and spine morphology via other than TrkB-dependent pathways, mainly via integrin signaling (*25*, *32*, *58*, *78*).

### MMP-9 cleaves proBDNF and is important for TrkB activation

To date, only a few substrates of MMP-9 have been identified in the brain. Mostly, these are transmembrane proteins and cell adhesion molecules, such as ICAM-5 (*79*), β-dystroglycan (*33*), nectin-3 (*34*), CD44 (*80*), or neuroligin-1 (*81*). Similarly, MMP-9 has been implicated in the cleavage of proBDNF into its mature form (mBDNF) (*20*, *21*). In Hwang *et al.* (*20*), incubation of the membrane fraction of cortical culture overexpressing proBDNF with a high concentration of recombinant MMP-9 caused BDNF maturation. Additionally, Mizoguchi *et al.* (*21*) have shown that repeated injections of mice with seizure-inducing pentylenetetrazole for several days increase the proportion of mBDNF to proBDNF, and this effect was diminished in MMP-9 KO animals. Our present study provides direct and complementary insights into the cleavage of proBDNF to mBDNF by MMP-9. Our cell-free assay provided an evidence that this process can occur in the absence of other proteases or the regulation of endogenous MMP-9 inhibitors such as TIMPs, which are present in tissues and block MMP-9 activity. MMP-9 is a part of a complex proteolytic system that exists outside of the cell and is known to affect synaptic plasticity (*19*, *23*, *25*). It is produced as a zymogen and needs proteolytic activation, for example, by plasmin (*24*). Therefore, there is a possible cascade where released tPA converts plasminogen to plasmin, which in turn can process proMMP-9 to active MMP-9. Plasmin, as well as MMP-9 (Fig. 5, G and H), converts proBDNF to mBDNF (*20*, *21*, *23*). Finally, both plasmin and MMP-9 are additionally regulated by their inhibitors, PAI-1 and TIMP1, respectively. It is possible that all, or only some parts of this cascade, exist around a single dendritic spine. Regardless of whether proBDNF is cleaved intracellularly by furin or extracellularly by plasmin, there is always a remaining N-terminal propeptide, which is known to activate p75^NTR^ receptor and facilitate LTD (*17*). It is also possible that MMP-9 cleaves proBDNF propeptide at different sites than furin and plasmin, making BDNF propeptide less efficient in activating p75^NTR^. Our Western blot data (Fig. 5G) might support this notion, since incubation with active MMP-9 produces also bands with higher molecular weight than mBDNF. Therefore, MMP-9 might mediate TrkB activation by degradation of BDNF propeptide and thus by reducing competing signaling from the p75^NTR^ receptor. Our data show that impairing MMP-9 activity by either application of chemical inhibitor or gene KO leads to a decreased level of TrkB activation during sLTP. While under the control condition, the receptor activation is sustained (~10 min after activation) (*40*), under an MMP-9 inhibition, it reverses back to the baseline.

Moreover, the role of BDNF in the plasticity-related effects has also been previously shown. Mizoguchi *et al.* (*21*) have shown that bilateral microinjections of pro-BDNF into the hippocampal dentate gyrus before each pentylenetetrazole treatment significantly enhanced kindling development in WT mice, but not in MMP-9 KO mice. Additionally, the effect of BDNF on neuronal activity–dependent increase in expression of MMP-9 has also been described (*82*).

**Fig. 6.**
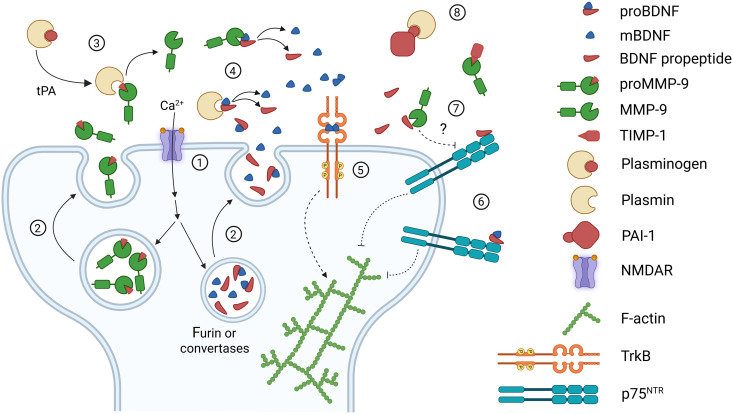
Proposed model of MMP-9 and BDNF interaction during synaptic plasticity. Activation of NMDAR (1) leads to the release of MMP-9 and BDNF (2), which might be released in its either pro-form or mature form with a propeptide. (3) tPA activates plasminogen to plasmin, which can also activate proMMP-9. (4) Plasmin and MMP-9 can extracellularly process proBDNF to mBDNF, which activates its receptor—TrkB (5). TrkB activation, together with other intracellular signaling, leads to the LTP cascade, including actin polymerization and cytoskeleton remodeling causing spine enlargement. (6) ProBDNF and BDNF propeptide, which is also co-released with mBDNF, can activate p75^TNR^, leading to LTD. (7) It is possible that MMP-9 can reduce bioactive BDNF propeptide and promote a competing TrkB activation. (8) Both plasmin and MMP-9 are blocked by their inhibitors, which control their action. Created in BioRender. Kalita, K. (2025) https://BioRender.com/nbak5x7.

Overall, our data indicate the pivotal role of MMP-9 in synaptic plasticity and associated TrkB activation, potentially through proBDNF processing to mBDNF. We demonstrate that a readily releasable pool of MMP-9, which is present at the dendritic spine, can be rapidly (seconds) released and the enzyme extracellular activity can lead to fast maturation of BDNF (summarized on Fig. 6). Additionally, our data may provide a functional link between MMP-9, BDNF and their involvement in many brain pathologies, which has previously been implicated, for example, in addiction (*83*), schizophrenia (*84*, *85*), ischemic stroke (*86*), or even cochlear implantation (*87*).

## MATERIALS AND METHODS

### Animals

All animal procedures were approved by the Max Planck Florida Institute for Neuroscience and Use Committees and were conducted in accordance with the National Institutes of Health (NIH) Guide for the Care and Use of Laboratory Animals, as well as with the Animal Protection Act of Poland (directive 2010/63/EU). Here, we used 4- to 8-day-old mice of either sex. C57BL/6J mice were obtained from Charles River, and MMP-9 KO mice [B6.FVB(Cg)-Mmp9tm1Tvu/J] were purchased from the Jackson Laboratory. MMP-9 KO mice were maintained by mating heterozygotes with heterozygotes. The genotype of each animal used was verified before preparing slices using polymerase chain reaction (PCR) of genomic DNA isolated from the tail. For experiments, we used MMP-9 KO mice and their WT littermates.

### Plasmids

The following plasmids were used: pCAG_GFP, pCAG_mCherry, pCAG_BDNF-SEP (subcloned from a pCMV_BDNF-SEP plasmid) (Addgene #83955), pCAG_MMP-9 [full-length mouse (MMP-9) from pcDNA3.1_MMP-9-HA] (Addgene #121172) (*88*) cloned into pCAG vector, and pCAG_MMP-9a1205c (full-length mouse MMP-9 with point mutation in single-nucleotide 1205 from adenine to cytosine resulting in codon change E402A, which results in the lack of MMP-9 enzymatic activity). Primers used for mutagenesis were as follows: forward: 5′-TGGCAGCGCACGCGTTCGGCCATGC, reverse: 5′-GCATGGCCGAACGCGTGCGCTGCCA. Primers used for NEBuilder HiFi DNA Assembly were as follows: forward: 5′-tgtctcatcattttggcaagGTGGTGGAATTCATGAGTC, reverse: 5′-tgctcaccatAGCGTAATCTGGAACATC. pCAG_MMP-9a1205c-SEP was obtained by amplification of SEP with PCR and cloning it into the pCAG_MMP-9a1205c vector using NEBuilder HiFi DNA Assembly. The following primers were used: forward: 5′-CCATACGATGTTCCAGATTACGCTATGAGTAAAGGAGAAGAACTTTTCACTGG, reverse: 5′-GTTTAAACGGGCCCTCTAGACTCGAGCGGCCGCTTATTTGTATAGTTCATC. For pCAG_MMP9a1205c-Gamillus, Gamillus obtained by PCR from Addgene (#124837) was inserted in place of SEP using NEBuilder HiFi DNA Assembly. The following primers were used: forward: 5′-agattacgctATGGTGAGCAAGGGCGAG, reverse: 5′-ggcagagggaaaaagatccgGCAGAATTCTTACTTGTACAGCTCG. pCAG_mCherry-Tetx was obtained by Yasuda laboratory (*40*). The TrkB FRET sensor consists of two components: the donor, which is TrkB fused to GFP at the C terminus (encoded by the plasmid pCAG_TrkB-GFP; Addgene #83952), and the acceptor, which is the SH2 domain of PLC-γ1 fused with two mRFP1 molecules at its N- and C-termini (encoded by the plasmid pCAG_PLC-RFP; Addgene #83953).

### Recombinant, inactive MMP-9 (E402A) production

Expression of the previously described (*58*) recombinant non-active mutant of MMP-9(E402A) was performed using the Bac-to-Bac Baculovirus expression system, according to the manufacturer’s instructions (Thermo Fisher Scientific). Briefly, DH10Bac competent cells were transformed with pFastBac1_MMP-9(E402A). Colonies that performed transposition of recombinant plasmid fragment into bacmid DNA were identified by blue-white selection, and recombinant bacmid was isolated and verified by PCR. The Sf21 insect cells were transfected with recombinant bacmid using Cellfectin reagent (Thermo Fisher Scientific) to obtain recombinant baculovirus. After amplification and titration of the recombinant baculovirus, High-Five cells were infected and incubated in the Sf-900IISFM serum-free medium (Thermo Fisher Scientific). Conditioned medium was collected 48 hours after infection, the medium was collected, and MMP-9(E402A) was purified by affinity chromatography with gelatin–Sepharose 4B (Cytiva) as previously described (*89*). Protein concentrations in the collected fractions were measured using Bradford reagent (Sigma-Aldrich).

### Rat hippocampal cultures

Dissociated hippocampal cultures from P0 (postnatal day 0) Wistar rats were prepared as previously described (*90*). Hippocampi were isolated on ice in dissociation medium (DM) [81.8 mM Na_2_SO_4_, 30 mM K_2_SO_4_, 5.8 mM MgCl_2_, 0.25 mM CaCl_2_, 1 mM Hepes (pH 7.4), 20 mM glucose, 1 mM kynureic acid, 0.001% phenol red] and were later incubated twice for 15 min at 37°C with 100 U of papain (Worthington, NY) in DM and rinsed three times in DM and subsequently three times in plating medium [minimum essential medium (MEM), 10% fetal bovine serum (FBS); Thermo Fisher Scientific]. Hippocampi were triturated in plating medium. Cells were diluted 1:10 in Opti-MEM (Thermo Fisher Scientific) and centrifuged for 5 min at room temperature at 200*g*. The resulting cell pellet was suspended in plating medium, and cells were counted in 1:1 dilution of 0.4% Trypan Blue solution (Thermo Fisher Scientific) and plated at a density of 150,000 cells per 18-mm-diameter coverslip (Assistent, Germany, #1.5) coated with poly-dl-lysine (1 mg/ml) (Sigma-Aldrich, P9011) and laminin (2.5 μg/ml) (Sigma-Aldrich, L2020). After 3 hours, plating medium was exchanged for maintenance medium (Neurobasal-A without phenol red, 2% B-27 supplement, 0.5 mM GlutaMAX, 25 μM β-mercaptoethanol; Thermo Fisher Scientific) and cells were kept at 37°C under a humidified 5% CO_2_ atmosphere. Cells were transfected with plasmids using Lipofectamine 3000 (Thermo Fisher Scientific) at 7 to 8 days in vitro (DIV). Lipofectamine-DNA complexes were prepared according to the manufacturer’s instructions and incubated with cells for 1 hour in the incubator, in fresh transfection medium (MEM without phenol red, 2% B-27 supplement, 1 mM pyruvate, 0.5 mM GlutaMAX, 25 μM β-mercaptoethanol; Thermo Fisher Scientific).

### Expansion microscopy

Rat hippocampal neuronal cultures were transfected with plasmids encoding tdTomato (for visualizing dendritic spine morphology), together with plasmids for either MMP-9–SEP or BDNF-SEP. After the paraformaldehyde fixation, samples were labeled with antibodies against mCherry (made in rat, Thermo Fisher Scientific, #M11217; 1:1000) and GFP (made in chicken, Thermo Fisher Scientific, #A10262; 1:1000), followed by incubation with secondary antibodies (Alexa Fluor 546 anti-rat antibody, Thermo Fisher Scientific #A11081, 1:500 and Alexa Fluor 488 anti-chicken antibody, Thermo Fisher Scientific #A11039, 1:500), and expanded with previously published protocol (*47*) and imaged with a confocal microscope (Cell Discoverer 7, 50× objective with Airyscan 2). Images of vesicles and neuronal morphology were then reconstructed with Imaris Image Analysis Software. For each sample, an expansion factor was determined by dividing the average area of nuclei (stained with Hoechst 33342; 0.1 μg/ml final concentration; Thermo Fisher Scientific #62249) after expansion by that before expansion. Vesicle size was subsequently adjusted based on this expansion factor.

### Total internal reflection fluorescence imaging

Rat hippocampal cultures at 7 DIV were transfected with pCAG_BDNF-SEP or with pCAG_MMP-9a1205c-SEP to image the release of BDNF and MMP-9, respectively. The cultures (19 to 21 DIV) were imaged in TIRF microscope Axio Observer.Z1 (Zeiss) equipped in a 488-nm laser for GFP excitation, alpha Plan-Apochromat 100×/1.46 Oil DIC M27 objective (Zeiss), and EMCCD camera—QuantEM 512SC (Photometrics) with a 512 × 512 resolution and 16-μm pixel size. The microscope acquisition was controlled by AxioVision Release 4.8.2 (Zeiss). Cells on #1.5 coverslips were imaged in a perfusion chamber with parallel platinum wires for electrostimulation (Warner Instruments #RC-49MFS). The movies were recorded with 2 × 2 binning, which resulted in a 0.32 × 0.32–μm pixel size. The imaging frequency was 20 Hz with a 13-ms frame acquisition time. All cells were first imaged without stimulation—basal conditions for 1200 frames (60 s), and later, another movie of 2400 frames (120 s) was recorded with electrical stimulation. For electrical stimulation, we used Model 4100 Isolated High Power Stimulator (A-M Systems).The electrical stimulation started 4 s after the beginning of the second movie and lasted for 60 s. It consisted of packages of 20× 1-ms impulses at 83 Hz, which were repeated 30× in total at 0.5 Hz. Cells were imaged in a buffer (145 mM NaCl, 2.5 mM KCl, 10 mM glucose, 10 mM Hepes, 0.4 mM Trolox, 2 mM CaCl_2_, pH 7.4) in the presence of 50 μM picrotoxin and 10 μM NBQX.

Recordings were analyzed using Suite2p software (*91*), which enabled automatic detection of events and fluorescence extraction. Fluorescent traces were then further processed with a MATLAB script to extract information about the timing of event occurrence.

### Imaging of the DQ-gelatin fluorescence

Rat neuronal cultures were transfected with pSyn-tdTomato. During the experiment day, neurons were incubated in ES enriched with 2 mM MNI-caged l-glutamate, 1 μM tetrodotoxin (TTX), 4 mM CaCl_2_, and DQ-gelatin (30 μg/ml) and were kept at room temperature (24°C). Spines of tdTomato-expressing neurons were imaged sequentially up to two spines. Red channel was used for determining the volume change, and DQ-gelatin conversion was examined in a green channel.

### Hippocampal organotypic culture and transfection

Hippocampal organotypic cultures were prepared from 4- to 8-day-old mouse pups (either C57BL/6J or MMP-9 KO and their WT littermates). Hippocampi were dissected and sliced into 350-μm-thick transverse slices with a McIlwain tissue chopper and placed on PTFE membranes (Millicell; Merck, catalog no. PICMORG50). Cultures were kept in the air-medium interphase; medium was composed of minimal essential medium supplemented with 20% heat-inactivated horse serum, 12.9 mM d-glucose, 5.2 mM NaHCO_3_, 30 mM Hepes buffer, 2 mM MgSO_4_, 1 mM l-glutamate, 1 mM CaCl_2_, 0.075% ascorbic acid, and insulin (1 μg/ml). Half of the medium volume was exchanged every 2 to 3 days. After 8 to 13 days in culture, slices were transfected using the GeneGun (Bio-Rad) method (*92*). Gene gun bullets were prepared with plasmids pCAG_GFP, 30 μg DNA (structural change experiments); pCAG_MMP-9a1205cSEP and pCAG_mCherry ratio 1:1, 50 μg DNA; or pCAG_MMP9a1205cGamillus with pCAG_mCherry ratio 1:1, 50 μg DNA (MMP-9 release experiments). For experiments with blocked autocrine vesicular release by coexpression of TeTx, pCAG_MMP9a1205cSEP was mixed with pCAG_mCherry-IRES-TeTx 1:1, 50 μg of DNA, and pCAG_TrkB-GFP and pCAG_PLC-RFP in ratio 1:3, 50 μg DNA (TrkB activation experiment). Neurons expressing GFP and MMP-9 SEP/Gamillus with mCherry were imaged 1 to 7 days after transfection. Neurons expressing TrkB were imaged within 12 to 48 hours from the transfection. For the rescue experiment, slices from MMP-9 KO mice were cotransfected with the pCAG_GFP plasmid together with either pCAG_MMP-9 (full-length MMP-9) or pCAG_MMP-9a1205c (an inactive mutant, E402A), ratio 1:2, 50 μg DNA.

### Two-photon imaging and FLIM

Two-photon imaging and FRET measurements were performed using a custom-built two-photon fluorescence lifetime imaging microscope as previously described (*40*). GFP fluorophore was excited with 1.2- to 1.5-mW, 920-nm pulsating tunable laser (Ti-sapphire laser, Coherent, Chameleon). Emitted light was collected through 60× water immersion objective (numerical aperture 0.9, Olympus), split with a dichroic mirror (565 nm). Photons were detected by two separate photoelectron multiplier tubes (PMTs; H7422-40p, Hamamatsu). For the green channel, light arriving to the PMT was filtered with a band-pass filter (510/70 nm, Chroma), and for red channel using band-pass filter (620/90 nm, Chroma). Fluorescence lifetime images were obtained using a time-correlated single-photon counting board (Time-harp 260, Pico-Quant) and processed by custom software FLIMage (https://github.com/ryoheiyasuda/FLIMage_public). Images for volume change measurements and TrkB activation were collected at 128 × 128 pixels with 7.8-Hz rate with 24-frame averaging. For MMP-9 SEP release imaging, 64 × 64–pixel frames were collected at a 7.8-Hz rate with no averaging.

### Two-photon glutamate uncaging

A second Ti-sapphire tunable laser (Coherent, Cameleon) was set to 720 nm and used to uncage MNI-caged l-glutamate (Tocris). Up to four spines in separate regions of interest (ROIs) per cell were stimulated simultaneously on four distinct secondary apical dendrites. Stimulation protocol consists of trains of 6-ms, 2.7- to 3-mW pulses (30 times at 0.5 Hz) near spines of interest.

Organotypic hippocampal slices were imaged in Mg^2+^-free artificial cerebrospinal fluid (ACSF) solution (127 mM NaCl, 2.5 mM KCl, 4 mM CaCl_2_, 25 mM NaHCO_3_, 1.25 mM NaH_2_PO_4_, and 25 mM glucose) containing 1 μM TTX (Tocris) and 2 mM MNI-caged-l-glutamate buffered with carbogen (5% CO_2_ and 95% O_2_). Experiments were performed at room temperature (24°C).

### Spine volume change measurements

Spines of GFP-expressing pyramidal neurons in CA1 subfield of hippocampus were imaged sequentially up to four spines located on separate secondary apical dendrites. For treatment, slices were perfused in ACSF (see above) with either DMSO (for control; final concentration, 0.08%) or MMP-9/MMP-13 Inhibitor I (catalog no. 444252, Calbiochem) in DMSO final concentration 5 μM for 30 min before the start of sLTP protocol. Experiments were repeated on organotypic cultures from at least three different dissections.

### Two-photon FLIM data analyses

The analysis of TrkB sensor activation was performed as previously described (*40*). To measure the fraction of a donor bound to an acceptor, we fit a fluorescence lifetime curve summing all pixels over a whole image with a double exponential function convolved with the Gaussian pulse response function

$$F\left(t\right)=F_{0}\left[P_{D}H\left(t,t_{0},\tau_{D},\tau_{G}\right)+P_{\text{AD}}H\left(t,t_{0},\tau_{\text{AD}},\tau_{G}\right)\right]$$

where τ_AD_ is the fluorescence lifetime of donor bound with acceptor; *P*_D_ and *P*_AD_ are the fraction of free donor and donor bound with acceptor, respectively; and *H*(*t*) is a fluorescence lifetime curve with a single exponential function convolved with the Gaussian pulse response function

$$H\left(t,t_{0},\tau_{D},\tau_{G}\right)=\frac{1}{2}\text{exp}\left(\frac{\tau_{G}^{2}}{2\tau_{D}^{2}}-\frac{t-t_{0}}{\tau_{D}}\right)\text{erfc}\left(\frac{\tau_{G}^{2}-\tau_{D}\left(t-t_{0}\right)}{\sqrt{2}\tau_{D}\tau_{G}}\right)$$

in which τ_D_ is the fluorescence lifetime of the free donor, τ_G_ is the width of the Guassian pulse response function, *F*_0_ is the peak fluorescence before convolution, *t*_0_ is the time offset, and erfc is the error function. We fixed τ_D_ to the fluorescence lifetime obtained from free eGFP (2.6 ns). To generate the fluorescence lifetime image, we calculated the mean photon arrival time, 〈*t*〉, in each pixel as

〈t〉=∫tF(t)dt/∫F(t)dt

then, the mean photon arrival time is related to the mean fluorescence lifetime, 〈τ〉, by an offset arrival time, *t*_0_, which is obtained by fitting the whole image

$$\langle \tau \rangle =\langle t\rangle -t_{0}$$

For small regions of interest (ROIs) in an image (spines or dendrites), we calculated the binding fraction (*P*_AD_) as follows

$$P_{\mathrm{AD}}=\tau_{D}\left(\tau_{D}-\langle \tau \rangle \right){\left(\tau_{D}-\tau_{\text{AD}}\right)}^{-1}{\left(\tau_{D}+\tau_{\text{AD}}-\langle \tau \rangle \right)}^{-1}$$

### TrkB activation

For TrkB activation, images were collected simultaneously for up to four ROIs located on distinct secondary apical dendrites of CA1 pyramidal neurons coexpressing TrkB-GFP and PLC-RFP constructs. Cells included in experiment had an average initial binding fraction not higher than 45%. Slices were treated with either DMSO or Inhibitor I as described above. Experiments were repeated on organotypic cultures from at least three different dissections. Binding fraction (*P*_AD_) was normalized by subtracting the averaged baseline binding fraction (*P*_AD0_; before sLTP protocol) with the formula *P*_AD_ − *P*_AD0_.

### MMP-9 release two-photon imaging

Spines were imaged sequentially from distinct proximal secondary dendrites of pyramidal cells in CA1 coexpressing MMP9-SEP and mCherry or MMP-9–Gamillus and mCherry. Spine volume change was calculated using the formula Δ*F*/*F*_0_ = (*F* − *F*_0_)/*F*_0_, where *F* is the sum of fluorescence in a given time from the ROI containing the spine of interest and *F*_0_ represents the average of *F* from the baseline of first five to seven time points (before sLTP protocol).

MMP-9 SEP release was calculated using traces from 13 spines, where normalized intensity was calculated using the formula Δ*F*/*F*_0_ = (*F* − *F*_0_)/*F*_0_, where *F*_0_ corresponds to an average of frames preceding the uncaging pulse and frames of uncaging pulse. Later, the results from uncaging pulses 2 to 18 were averaged. The same analysis was performed for data collected from red channel corresponding to cytoplasmic mCherry treated as spine volume.

To assess the fast kinetics of MMP-9 release following uncaging pulses, we analyzed fluorescence signals within 2 s between uncaging pulses. We divided fluorescence data of the stimulated spine into 16-frame sweeps (corresponding to 2.048-s uncaging intervals). We aligned the data so that each uncaging pulse occurred between the second and the third frame (the second row on heatmap) in each sweep (fig. S3). Then, the fluorescence data were normalized to the average fluorescence of the first two frames in each sweep. This analysis allowed us to visualize the averaged fluorescence changes normalized to fluorescence before each uncaging pulse to avoid the cumulative effect of release and spine volume increase (averaged over 2 frames) as a heatmap (fig. S3). Visually brighter pixels in the heatmap, indicating increased fluorescence, appeared between 16th and 56th second of imaging, corresponding to the 2nd to the 22nd uncaging pulses. After this period, there were no detectable release events (fig. S3). Therefore, we averaged SEP and (separately) mCherry fluorescence in sweeps between 2nd and 18th uncaging pulse and plotted them next to each other. The same analysis was conducted for MMP-9–Gamillus experiment.

### proBDNF digestion assay

Twenty nanograms of recombinant proBDNF (Alomone Labs) was incubated with 50 ng of recombinant MMP-9 (Calbiochem) or 50 ng of recombinant, human, inactive MMP-9 (E402A) in total volume of 20 μl. All reactions were incubated in reaction buffer (final concentration: 150 mM NaCl, 10 mM CaCl_2_, 0.01% bovine serum albumin, 50 mM tris-Cl, pH 7.5) for 16 hours at 30°C.

### Western blotting

ProBDNF digestion samples were run on 15% SDS–polyacrylamide gel electrophoresis (PAGE) gels and electrotransferred onto polyvinylidene difluoride membrane (Immobilon-P, Millipore) using the semi-dry method. Membranes were blocked for 2 hours at room temperature with 10% (w/v) dried nonfat milk powder in tris-buffered saline with 0.1% Tween 20 (TBS-T). After blocking, the membranes were incubated at 4°C overnight with rabbit anti-BDNF antibody (Santa Cruz Biotechnology) diluted 1:500 in 5% (w/v) nonfat milk in TBS-T. Membranes were then incubated for 2 hours at room temperature with horseradish peroxidase–labeled secondary antibody (Goat Anti-Rabbit IgG Antibody; Vector Laboratories) diluted 1:10,000 in 5% dried nonfat milk powder in TBS-T. After washing, the peroxidase activity was visualized on photographic film with ECL Prime Western Blotting Detection Reagent (Cytiva). The developed film was later digitized on ChemiDoc MP Imaging System (Bio-Rad) and analyzed using Image Lab software (Bio-Rad).

### Statistical analysis

All values are presented as means ± SEM unless stated otherwise. The number of independent measurements [*n*(spines/neurons)] is indicated in figure legends. Unpaired two-tailed Student’s *t* test was used for comparing two independent samples. Paired two-tailed Student’s *t* test was used for matched before-after measurements of vesicular release or matched mCherry and MMP-9–SEP or MMP-9–Gamillus signals. Repeated-measures two-way analysis of variance (ANOVA), followed by multiple comparison tests, was used to compare grouped datasets of time-lapse data (Prism 10, GraphPad). Data were excluded from analysis if dendritic bleeding, spine collapse, or any other obvious signs of cellular health were apparent.

### Figure preparation

All figures were prepared using Inkscape 1.3.2 (www.inkscape.org).
