# Supplementary material for: BDNF-driven synaptic plasticity requires autocrine matrix metalloproteinase–9 activity
Source: Sci Adv. 2025 Sep 24;11(39):eadx2369. doi: 10.1126/sciadv.adx2369 (PMC12459465; doi:10.1126/sciadv.adx2369)
Supplement: Supplementary file 1 — Figs. S1 to S4 Legends for movies S1 to S6 [file sciadv.adx2369_sm.pdf]

Science Advances
AAAS

# Supplementary Materials for

## BDNF-driven synaptic plasticity requires autocrine matrix metalloproteinase–9 activity

Diana Legutko *et al.*

Corresponding author: Piotr Michaluk, p.michaluk@nencki.edu.pl; Ryohei Yasuda, ryohei.yasuda@mpfi.org; Leszek Kaczmarek, l.kaczmarek@nencki.edu.pl

*Sci. Adv.* **11**, eadx2369 (2025)
DOI: 10.1126/sciadv.adx2369

**The PDF file includes:**

Figs. S1 to S4
Legends for movies S1 to S6

**Other Supplementary Material for this manuscript includes the following:**

Movies S1 to S6

**Fig. S1.**

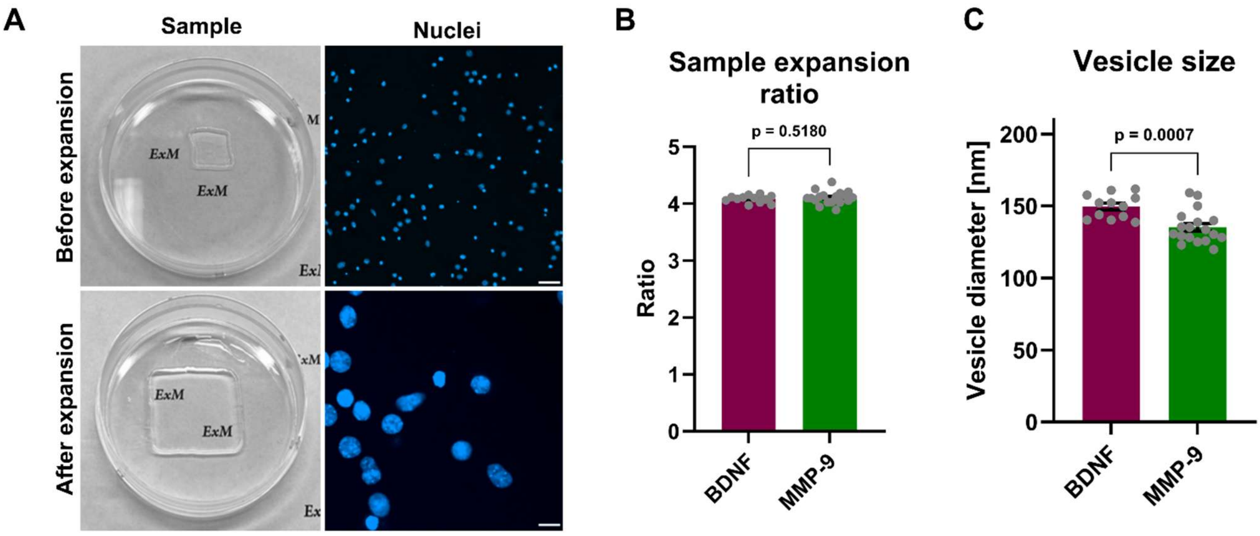


**Fig. S1: Expansion factor measurements and size of BDNF-SEP and MMP-9-SEP vesicles. A)** Images of Hoechst-stained nuclei and gel sample before (upper panel) and after (lower panel) the expansion. **B)** Graph representing sample expansion ratio, measured as the ratio of nuclei size before and after the expansion procedure. Data are means ± SEM and dots represent average values for individual nuclei. BDNF-SEP (n = 12 images, 12 cells), MMP-9-SEP (n = 18 images, 18 cells). Unpaired t-test ($t = 0.6547$, $df = 28$, $p = 0.5180$). **C)** Graph representing size of vesicles with either BDNF-SEP or MMP-9-SEP. Actual size of vesicles was corrected by the expansion factor measured as change of nuclei size. Data are means ± SEM and dots represent average values for individual neurons. BDNF-SEP (n = 364 vesicles, 12 images, 12 cells), MMP-9-SEP (n = 451 vesicles, 18 images, 18 cells). Unpaired t-test ($t = 3.823$, $df = 28$, $p = 0.0007$).

**Fig. S2.**

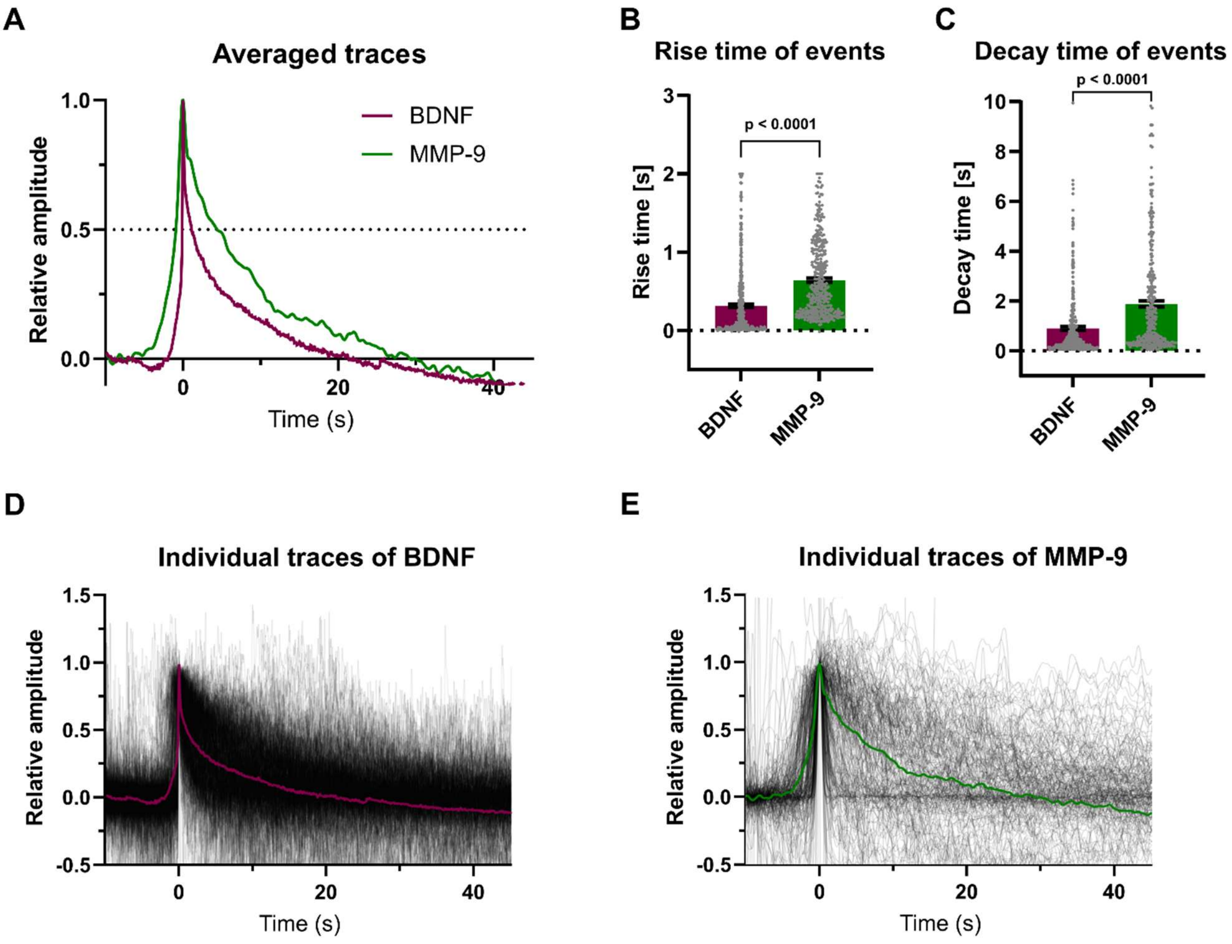


**Fig. S2. Kinetic properties of BDNF-SEP and MMP-9-SEP vesicles. (A)** Averaged traces of all recorded BDNF-SEP (magenta) and MMP-9-SEP (green) transients evoked by electrical stimulation of the neuronal cultures. Transients were time-adjusted to their peaks, and the amplitude is normalized from 0 to 1. **(B)** Rise times of recorded events evoked by electrical stimulation of the neuronal cultures. Data are means ± SEM and dots represent individual values. BDNF-SEP (n = 366 events, 28 cells), MMP-9-SEP (n = 251 events, 72 cells). Mann-Whitney test ($p < 0.0001$). (**C**) Decay times of recorded exocytosis events evoked by electrical stimulation of the neuronal cultures. All markings and "n" values are as in (**B**). Mann-Whitney test ($p < 0.0001$). **(D)** Superimposed individual traces of released BDNF-SEP (n = 366 traces). Purple line – averaged trace. **(E)** Superimposed individual traces of released MMP-9-SEP (n = 251 traces). Green line – averaged trace.

**Fig. S3.**

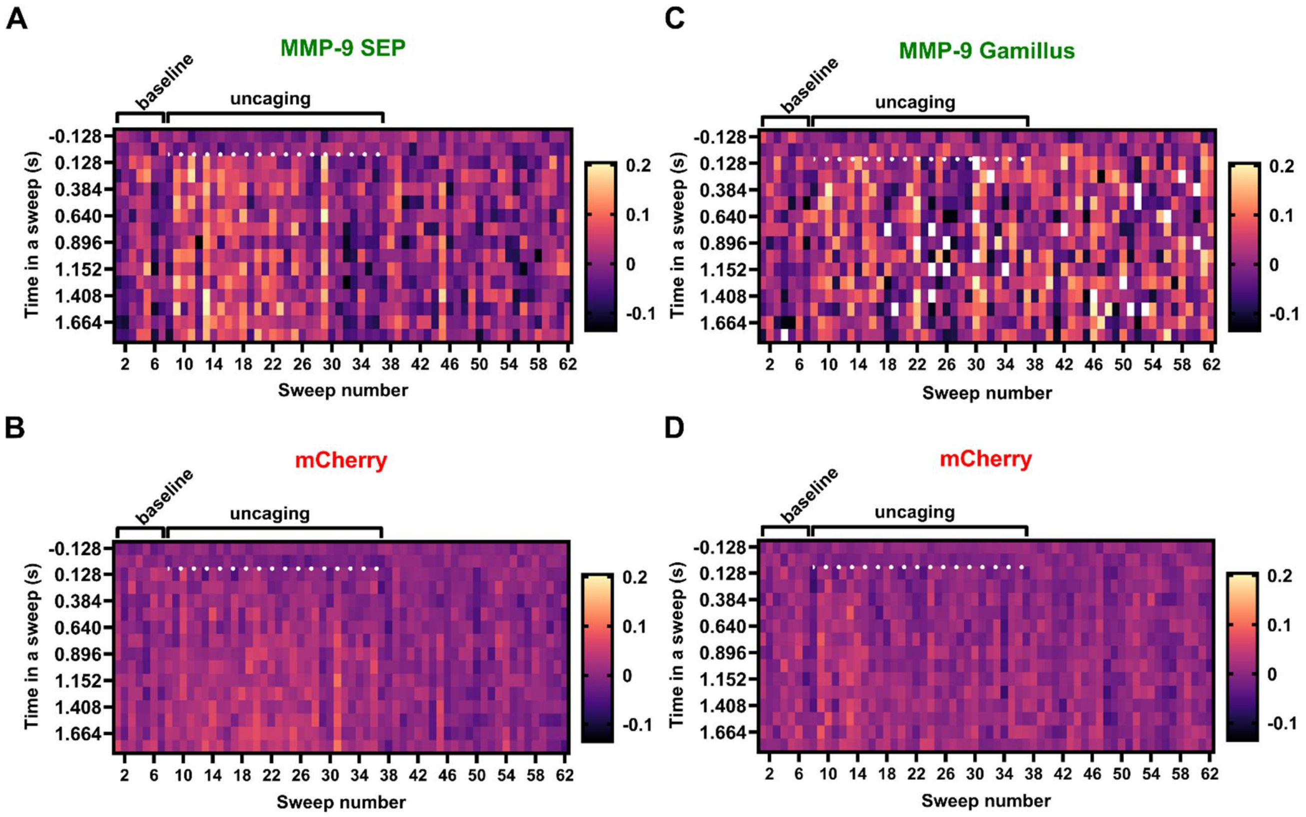


**Fig. S3. Analysis of MMP-9 SEP fluorescence between uncaging pulses.** (**A**) Heatmap representing averaged fluorescence of MMP-9-SEP in stimulated spines (in the DMSO condition). Each colored rectangle represents relative fluorescence intensity of SEP in a stimulated spine. Imaging data were divided into single trials (sweeps – columns) of 16 frames matching uncaging frequency, so that uncaging pulse always occurs in a second frame of the sweep. Each sweep (column) is normalized to the baseline which corresponds to first two frames of the sweep. sLTP protocol starts after recording baseline of 7 sweeps (~ 14 s) and lasts for 60 seconds (30 pulses, 0.5 Hz). White dots represent uncaging laser pulse which occurs always between the second and the third frame in a sweep during sLTP-evoking protocol. (**B**) Heatmap representing averaged fluorescence of mCherry in stimulated spines (in the DMSO condition) corresponding to the MMP-9 SEP signal shown in panel (**A**). All marking is the same as in panel (A). (**C**) Control experiment using MMP-9 fused with Gamillus (pH-stable form of GFP). Heatmap represents averaged fluorescence of MMP-9-Gamillus in stimulated spines (in the DMSO condition). All markings is the same as in panel (A). (**D**) Heatmap representing averaged fluorescence of mCherry in stimulated spines (in the DMSO condition) corresponding to the MMP-9 Gamillus signal shown in panel (C). All marking is the same as in panel (A).

**Fig. S4.**

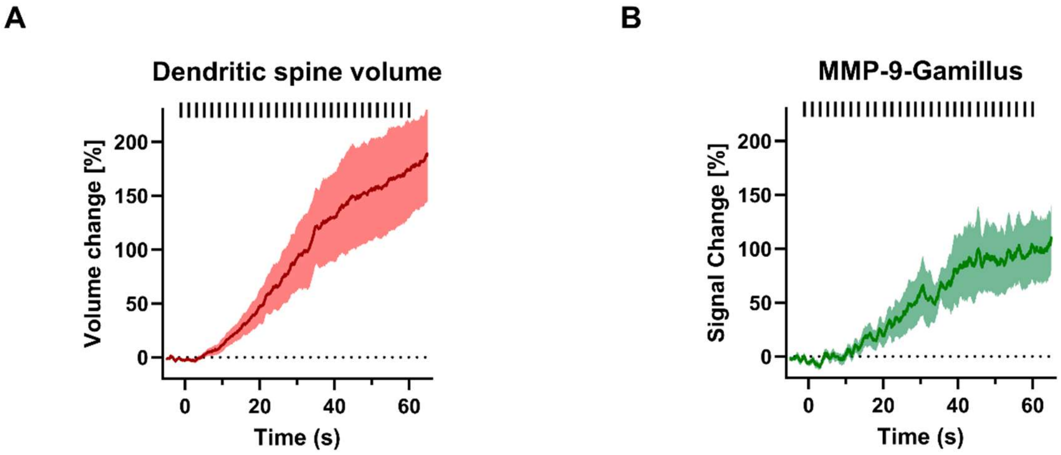


**Fig. S4. Spine volume changes and a release of MMP-9-Gamillus. (A)** Averaged time courses of changes in fluorescence of mCherry measured as $\Delta F/F_0$ for spines stimulated by uncaging. Data are means (red line) ± SEM (shading). Timing of glutamate uncaging laser pulses during sLTP protocol are indicated by black bars above the graph. (n = 13 spines, 5 cells). **(B)** The same as in (A) but for corresponding MMP-9-Gamillus fluorescence.

**Movie Captions**

**Movie S1.**

**BDNF-SEP release.** A video showing TIRF microscopy recording of BDNF-SEP release. Each frame lasts 50 ms and the movie is accelerated 2 times. After frame 200 there is an appearing "Stim." sign indicating electrical stimulation. Additionally, there are appearing yellow rings around individual exocytosis events.

**Movie S2.**

**MMP-9-SEP release.** A video showing TIRF microscopy recording of MMP-9-SEP release. Each frame lasts 50 ms and the movie is accelerated 2 times. After frame 200 there is a appearing "Stim." sign indicating electrical stimulation. Additionally, there are appearing yellow rings around individual exocytosis events.

**Movie S3.**

**MMP-9 gelatinolytic activity.** Two-photon microscopy video demonstrating the increase of signal from DQ™ gelatin, a fluorogenic gelatin substrate that emits green fluorescence when cleaved by metalloproteinases (upper panel) and corresponding signal cytoplasmic fill (lower panel). The video is in 7 Hz and images were acquired in a single plane at 7.8 Hz frequency. Uncaging pulses were delivered every 16 frames for 30 times. Time of uncaging pulse can be visible as a yellow cross on the top for the frame. Red signal represents fluorescence of mCherry cytoplasmic fill and green signal represents MMP-9-SEP.

**Movie S4.**

**MMP-9 release during structural plasticity**. Two-photon microscopy video demonstrating the increase of MMP-9-SEP fluorescence during the uncaging protocol. The video is in real time. Images were acquired in a single plane at 7.8 Hz frequency. Uncaging pulses were delivered every 16 frames for 30 times. Time of uncaging pulse can be visible as a red bar on the top for the frame. Red signal represents fluorescence of mCherry cytoplasmic fill and green signal represents MMP-9-SEP.

**Movie S5.**

**Spine-head enlargement during structural plasticity.** Two-photon microscopy video demonstrating the volume increase upon uncaging during structural plasticity experiment. The images (maximal projections of Z-stacks) were acquired every minute before and after uncaging protocol. During the uncaging protocol (marked with appearing white dot) images were collected in a single plain at 7.8 Hz frequency. Time is indicated on the video.

**Movie S6.**

**TrkB activation during structural plasticity.** Two-photon FLIM video demonstrating activation of TrkB sensor. The images (maximal projections of Z-stacks) were acquired every minute before and after uncaging protocol. During the uncaging protocol (marked with appearing white dot) images were collected in a single plain at 7.8 Hz frequency. Time is indicated on the video. Warmer colors represent shorter GFP lifetimes corresponding to increased binding fraction and higher TrkB activity.
